# Venous hypertension caused by a meningioma involving the sigmoid sinus: case report

**DOI:** 10.1186/s12883-021-02144-5

**Published:** 2021-03-17

**Authors:** Koichiro Sumi, Naoki Otani, Fumi Mori, Shun Yamamuro, Hideki Oshima, Atsuo Yoshino

**Affiliations:** Division of Neurosurgery, Department of Neurological Surgery, Nihon University School of Medicine, 30-1 Oyaguchi-Kamimachi, Itabashi-ku, Tokyo, 173-8610 Japan

**Keywords:** Venous hypertension, Sigmoid sinus, Meningioma, Case report

## Abstract

**Background:**

Intracranial venous hypertension has been associated with a few cases of meningioma secondary to compression of the venous sinus. This is the rare case of small meningioma involving the sigmoid sinus leading to intracranial venous hypertension mimicking venous thrombosis.

**Case presentation:**

A 39-year-old woman suffered visual dysfunction due to bilateral papilledema. Noncontrast head computed tomography (CT) showed no intracranial space-occupying lesions or hydrocephalus. Cerebrospinal fluid examination revealed high opening pressure. Various image inspections such as three-dimensional CT angiography, magnetic resonance imaging, and cerebral angiography demonstrated a small 2.5-cm lesion causing subtotal occlusion of the dominant right sigmoid sinus. No improvement of clinical manifestations was observed after medical treatment for 6 months, so right presigmoid craniectomy was performed. Operative findings revealed that the tumor was located predominantly involving the sigmoid sinus. The pathological diagnosis was fibrous meningioma. Postoperative fundoscopic examination showed improvement of bilateral papilledema.

**Conclusions:**

We treated a patient presenting with intracranial hypertension due to a small meningioma involving the sigmoid sinus. This unusual case suggests that early surgical strategies should be undertaken to relieve the sinus obstruction.

## Background

Meningiomas are the most common type of extra-axial tumors of the meninges, and may occur at any location along the meninges encasing the central nervous system. Many meningiomas are commonly asymptomatic and found incidentally, but large meningiomas can manifest as symptoms of mass effect such as headache, focal neurological deficits, and seizures. Intracranial hypertension has been associated with a few cases of meningioma secondary to compression of the venous sinus [1–3]. Intracranial hypertension can be caused by impairment of blood flow due to occlusion or severe stenosis of the posterior superior sagittal sinus [4, 5], at the torcular herophili, sigmoid sinus, or only transverse sinus with significant dominance on one side [6–10].

We describe a rare case of intracranial hypertension caused by meningioma located inside the dominant sigmoid sinus.

## Case presentation

A 39-year-old woman presented to the ophthalmology department of our hospital with discomfort in her eyes. Fundoscopic examination showed bilateral papilledema. Extensive neuro-ophthalmologic examination revealed mild visual field abnormalities. She was referred to the neurosurgery department for suspected abnormally high intracranial pressure (ICP). Neurological examination found normal consciousness with no abnormalities. Her cranial nerves were intact. She had no diplopia, weakness, ataxia, or sensory disturbance. Coagulation profile and biological tests were within normal limits. She was overweight (body mass index of 27.2 kg/m^2^). Her medical and family history was unremarkable, and no evidence of infection was found including sinusitis, otitis media, and mastoid cellulitis. She was not taking any medication, including oral contraceptives or hormonal agents. She had previously experienced four normal vaginal deliveries (gravidity and parity G4P4) without complications. Cerebrospinal fluid (CSF) examination revealed high opening pressure (500 mmH_2_O [> 35 cm]) on lumbar puncture and normal CSF composition.

**Fig. 1:**
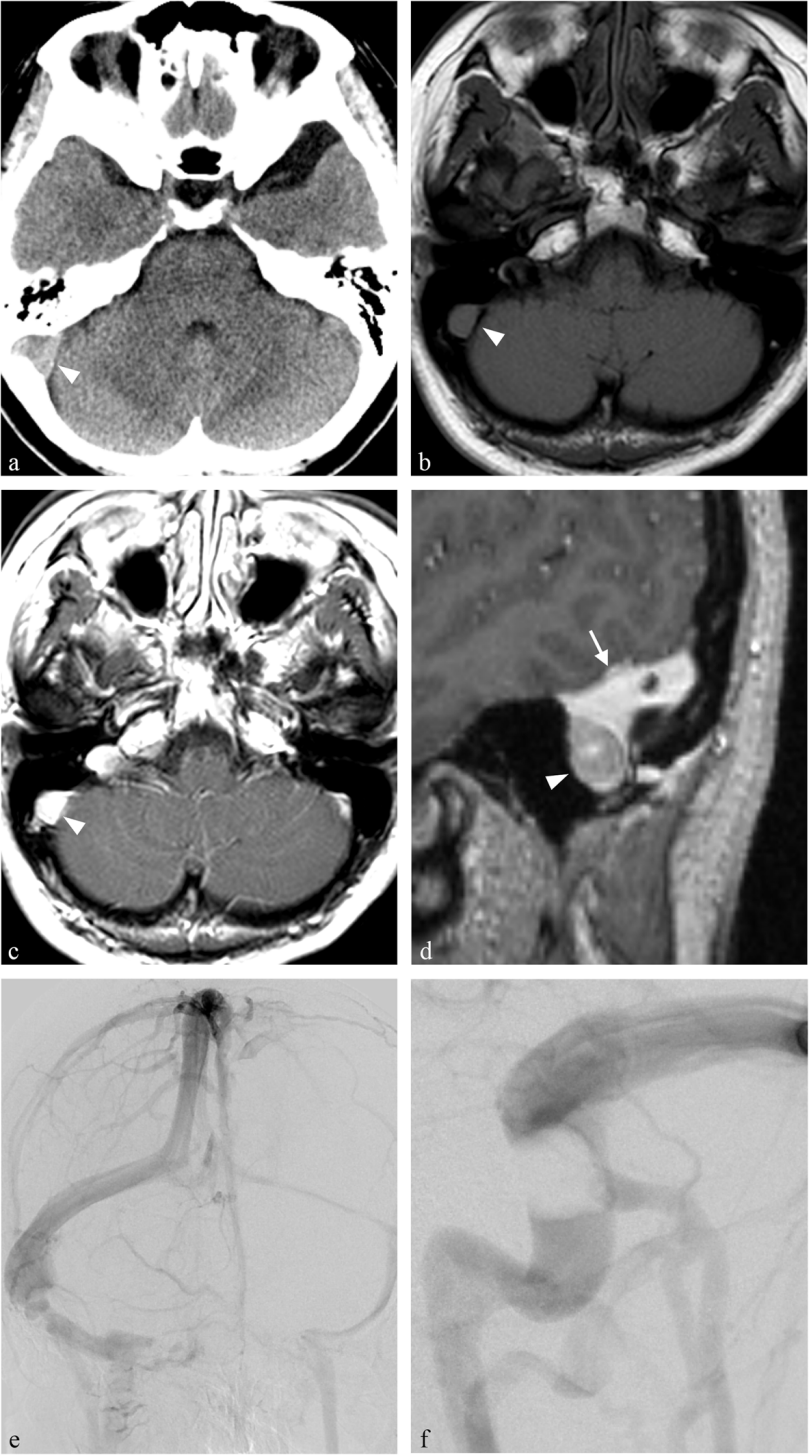
**a** Preoperative computed tomography (CT) scan revealing no intracranial space-occupying lesions or hydrocephalus except an asymptomatic arachnoid cyst of the left middle fossa. **b**–**c** Preoperative magnetic resonance images showing the lesion in the right sigmoid sinus (*arrowhead*) appearing as isointense on T1-weighted image (**b**), and with homogeneous enhancement following intravenous administration of gadolinium (**c**). No obvious dural tail sign is present. **d** Sagittal gadolinium-enhanced T1-weighted images revealing the mass lesion (*arrowhead*) located under the transverse sinus (*arrow*). Mass lesion causes severe luminal narrowing. **e** Conventional angiogram demonstrating the dominant right transverse sinus with hypoplastic left transverse sinus, and subtotal occlusion of the dominant right sigmoid sinus in the venous phase, with antegrade right transverse flow and poor collateral flow indicating this flow is dependent even in subtotal occlusion. **f** Conventional angiogram, venous phase, showing the mass lesion apparently located at the inner sinus wall mimicking venous thrombosis

Noncontrast computed tomography (CT) revealed no intracranial space-occupying lesions or hydrocephalus except an asymptomatic arachnoid cyst of the left middle fossa (Fig. 1a). The clinical manifestations and radiological findings indicated venous thrombosis. Magnetic resonance (MR) imaging revealed a small (2.5 cm) lesion located in the right sigmoid sinuses appearing as isointense on T1-weighted images, and isointense on T2-weighted images, with homogeneous enhancement following intravenous administration of gadolinium (Fig. 1b–d).

CT venography demonstrated subtotal occlusion of the right sigmoid sinus, caused by a well-defined, homogeneous, hypodense mass. Conventional angiography demonstrated the dominant right transverse sinus with hypoplastic left transverse sinus and subtotal occlusion of the dominant right sigmoid sinus in the venous phase, and a mass lesion causing severe luminal narrowing (Fig. 1e-f). We considered that this mass lesion interrupted the venous drainage, leading to venous hypertension. The precise location of the mass lesion was difficult to distinguish as the inner sinus wall and invading the sinus, or the extra sinus wall and compressing the sinus.

We investigated methods for improving blood flow by intravascular surgery such as stent placement and percutaneous transluminal angioplasty using a balloon. However, preoperative angiography showed antegrade right transverse flow and poor collateral flow indicating this flow is dependent even in subtotal occlusion. Therefore, sacrifice of the transverse or sigmoid sinus seemed to introduce critical risk. Surgical treatment or intravascular surgery may be high risk without knowing specific location of the mass lesion, inside or outside the sinus, and without knowing the pathological type. Therefore, our patient was treated with lumbar puncture followed by acetazolamide. Oral warfarin was initiated because it was unable to exclude the possibility of venous thrombosis. Frequent ophthalmologic assessments including quantitative visual fields were planned. We expected development of collateral flow and improvement of ICP, but unfortunately imaging showed no change. Slight deterioration of the bilateral papilledema was noted 6 months later. Lumbar puncture confirmed persistent elevation of CSF pressure with opening pressure of 420 mmH_2_O. We judged that improvement due to medical treatment could not be expected, so we planned surgical treatment.

**Fig. 2:**
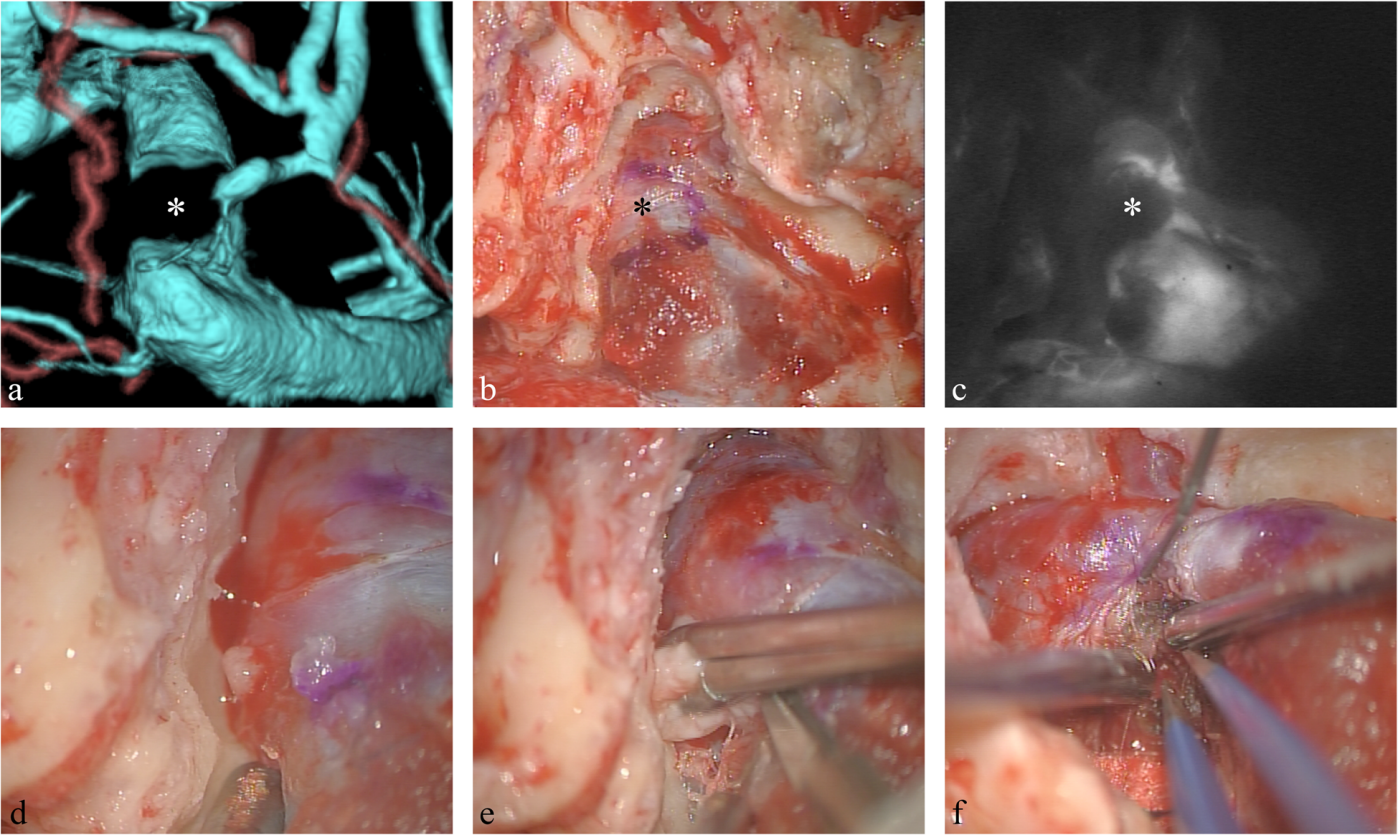
**a** Operative CT venogram demonstrating subtotal occlusion of the right sigmoid sinus, with severe luminal narrowing on the posterior side. The emissary vein was found downstream of the severe stenosis. The tumor (*asterisk*) is located inside the sigmoid sinus. **b** Right presigmoid craniectomy was performed. After drilling the petrosal bones, very high pressure was found in the transverse and sigmoid sinuses. **c** Intraoperative indocyanine green fluorescence angiogram revealing a mass lesion as a blood flow defect. The mass was located inside the sigmoid sinus. **d**–**f** The tumor was observed after retraction of the dura and sinus, without dura or sinus wall incision. The tumor was extruded out spontaneously, because of the high pressure in the sinus. The extra sinus part of the tumor was removed first, and then the tumor was followed into the sinus. The tumor was located predominantly involving the sigmoid sinus

Right presigmoid craniectomy was performed for tumor resection under motor evoked potential and somatosensory evoked response monitoring. The transverse and sigmoid sinuses were exposed by drilling of the petrosal bones. The transverse and sigmoid sinuses were elastic and hard, suggesting very high pressure in the sinuses (Fig. 2b). Intraoperative indocyanine green fluorescence angiography revealed the mass lesion as a blood flow defect in the sigmoid sinus (Fig. 2c). The tumor was directly observed after retraction of the dura and sinus. The tumor extruded out spontaneously without dura or sinus wall incision, because of the high pressure in the sinus. The extra sinus part of the tumor was removed first, and then the tumor was followed into the sinus. The tumor was located lateral sigmoid sinus, had invaded the sigmoid sinus, and attached to the sinus wall projected inside the sigmoid sinus. No attachment with the skull was found (Fig. 2d–f). Observation of the inside of the dura found no tumor on the inner layer of the dura or arachnoid membrane. Bleeding under high pressure occurred during resection of the intra-sinus tumor, which suggested the tumor was located in the sigmoid sinus and attached to the inside the sinus wall. Small residual tumor remained involving the transverse sinus, but the patency was checked immediately by Doppler probe or indocyanine green fluorescence.

Histological examination of the surgical specimen revealed spindle-shaped tumor cells, with narrow rod-shaped nuclei arranged in intersecting fascicles, without mitotic activity, nuclear atypia, or necrosis. The pathological diagnosis was fibrous meningioma World Health Organization grade I.

**Fig. 3:**
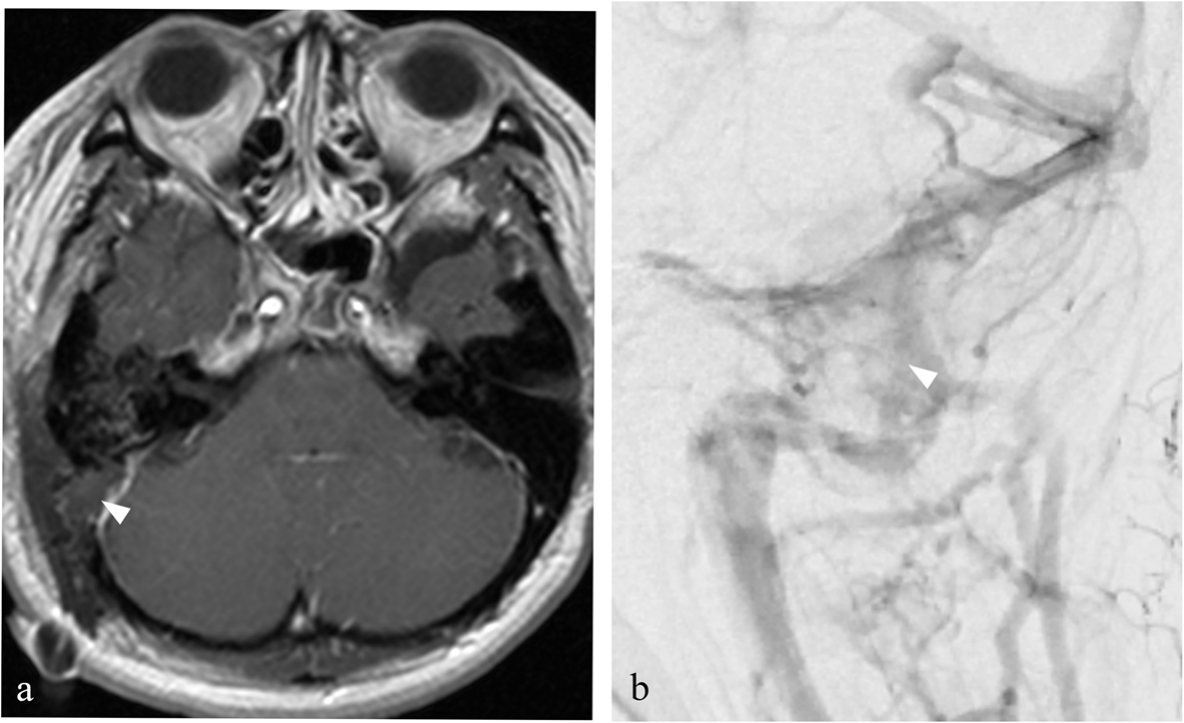
**a** Postoperative gadolinium-enhanced T1-weighted magnetic resonance images showing removal of the tumor (*arrowhead*). **b** Postoperative cerebral angiography showed patency of the left transverse and sigmoid sinuses showing removal of the tumor (*arrowhead*)

The patient’s postoperative course was uneventful. Postoperative MR imaging showed that the tumor was totally resected (Fig. 3a). Postoperative CT venography and cerebral angiography showed patency of both the right transverse and straight sinuses (Fig. 3b). Fundoscopic examination showed improvement of bilateral papilledema. Visual field testing and CSF examination found no abnormalities. The patient was initially negative about the surgery, but finally, she was glad to receive the surgery.

## Discussion and conclusion

Idiopathic intracranial hypertension (IIH) is a condition associated with increased ICP in the absence of intracranial pathological findings such as mass lesions or cerebral edema [11]. The cause of IIH is unknown but probably involves obstruction of the cerebral venous outflow [12, 13]. On the other hand, identifiable secondary causes included venous thrombosis [14] or tumor. Intracranial tumor compressing or invading the dural sinuses including meningioma [1, 4–9], solitary fibrous tumor/hemangiopericytoma [15], osteoma [16], osteoblastoma [17], epidermoid cyst [18], metastatic carcinoma [19, 20], Ewing sarcoma [21], and cholesteatoma [22] must be distinguished from venous thrombosis or IIH. Meningiomas are the most common neoplastic lesion causing venous hypertension.

In the present case, the differential diagnosis after non-contrast CT is venous thrombosis or IIH based on clinical presentation, ophthalmological evaluation for papilledema, and radiological findings of absence of intracranial space-occupying lesions, brain edema, or hydrocephalus. CT venography demonstrated filling defect in the dominant sigmoid sinus indicating venous thrombosis. The MR imaging findings of venous thrombosis are expected to show acute thrombus as isointense on T1-weighted images and hypointense on T2-weighted images, and subacute thrombus as hyperintense on T1- and T2-weighted images. Thrombus appears as prominent hypointense on susceptibility-weighted images [23] and T2*-weighted conventional gradient-echo images [24]. Gadolinium-enhanced T1-weighted imaging shows a filling defect in the sinus thrombosis. In the present case, the lesion appeared isointense on T1-weighted images with homogeneous enhancement following intravenous administration of gadolinium. However, various imaging methods such as three-dimensional CT angiography, MR imaging, and cerebral angiography could not identify the precise location inside or outside the sinus.

Meningioma sometimes invades the dural venous sinus. Meningiomas can be classified according to the degree of sinus invasion [25]: Type I, lesion attachment to the outer surface of the sinus wall; Type II, tumor fragment inside the lateral recess; Type III, invasion of the ipsilateral wall; Type IV, invasion of the lateral wall and roof; and Types V and VI, complete sinus occlusion with or without one wall free. However, the present tumor was attached to the roof of the sigmoid sinus, with possible extension into the sinus, and consequently is outside the classification. This case seems to correspond to type IV, but differs in the small or absence development on the inner surface of the dura.

Surgery is considered to carry high risk if the lesion location is unclear inside or outside the sinus, or if the tumor invades but does not completely obliterate the dominant transverse or sigmoid sinus, and outflow is strongly dependent on this sinus. Sacrifice of the sinus under such conditions can be a fatal complication such as hemorrhagic venous infarction, diffuse cerebral edema, seizures, or even death [25, 26]. Therefore, we chose medical treatment in anticipation of collateral circulation development. Meningioma is typically a slow-growing tumor, so venous invasion occurs very gradually. Most cases of venous invasion remain asymptomatic because of the development of venous collaterals. However, our patient showed no improvement of clinical manifestations after medical treatment for 6 months, indicating the collateral circulation was not fully developed. Eventually, surgical treatment was performed because of worsening of the congestive papilla.

We describe a rare case of intracranial venous hypertension due to a small meningioma causing obstruction of the dominant sigmoid sinus. This unusual case suggests that early surgical strategies should be undertaken to relieve the sinus obstruction.

## Data Availability

All relevant data related to this case report are contained within the present manuscript.

## Abbreviations

CSF: Cerebrospinal fluid
CT: Computed tomography;
MR: Magnetic resonance
ICP: Intracranial pressure
IHI: Idiopathic intracranial hypertension.
